# Palliative Performance Scale and survival in patients with cancer and non-cancer diagnoses needing a palliative care consultation: a retrospective cohort study

**DOI:** 10.1186/s12904-021-00773-8

**Published:** 2021-05-26

**Authors:** Patcharaporn Prompantakorn, Chaisiri Angkurawaranon, Kanokporn Pinyopornpanish, Lalita Chutarattanakul, Chanchanok Aramrat, Chanapat Pateekhum, Nisachol Dejkriengkraikul

**Affiliations:** Department of Family Medicine, Faculty of Medicine, Chiang Mai University, Chiang Mai, Thailand

**Keywords:** Palliative care, Palliative Performance Scale, Survival, Prognosis, Cohort study

## Abstract

**Background:**

Palliative Performance Scale (PPS) has been frequently used to estimate the survival time of palliative care patients. The objective was to determine the associations between the PPS and survival time among cancer and non-cancer patients in Thailand.

**Methods:**

This is a retrospective cohort study. All in-patient adults who received a palliative care consultation at Chiang Mai University Hospital between 1 July 2018 to 31 July 2019 were included in the study and were followed-up until 26 June 2020. The Palliative Performance Scale was assessed using the validated Thai-Palliative Performance Scale for Adults. Survival analysis was used to determine the association between the Palliative Performance Scale and survival time among cancer and non-cancer patients.

**Results:**

Out of 407 patients, 220 were male (54.1%). There were 307 cancer patients (75.4%) and 100 non-cancer patients (24.6%). The PPS and survival time in cancer patients were significantly correlated. Cancer patients with PPS 10, 20, 30, 40–60, and 70–80% had a median survival time of 2, 6, 13, 39, and 95 days, respectively. Non-cancer patients with PPS 10, 20, and 30% had a median survival time of 8, 6, and 24 days, respectively.

**Conclusions:**

While useful for estimating survival time for cancer patients, other factors should be taken into account in estimating the survival time for non-cancer patients.

**Supplementary Information:**

The online version contains supplementary material available at 10.1186/s12904-021-00773-8.

## Introduction

The World Health Organization (WHO) estimates that more than 56.8 million people require palliative care globally every year, with 25.7 million people being near their last days of life. The number of people in need of palliative care is increasing, particularly in low and middle-income countries, where access to palliative care is scarce [1]. When caring for palliative care patients, the diseases that patients face are advanced, making prognostication crucial for coordinating a treatment plan between patients, families, and medical teams [2, 3].

However, prognostication in palliative care is extremely challenging. The stage of cancer and progression of the disease do correlate with survival time in general, although they are not the only factors influencing survival [4]. Different types of diseases manifest in different disease trajectories at the final stages. For advanced cancer patients, patients may function relatively well for a long time and then rapidly decline until death. For non-cancer patients, the trajectories may vary. Patients with congestive heart failure or end-stage renal disease usually exhibit a gradual functional decline with frequent acute exacerbations, where every episode can potentially result in death. By contrast, demented or frail elderly patients usually exhibit a slow steady functional decline over a long period of time [5, 6]. Each distinct trajectory results in different utilization of palliative care and other medical care at the end of life [7]. It is necessary for doctors to accurately estimate the survival time promptly for both cancer and non-cancer patients to ensure that the appropriate treatment planning could be made for each individual, harm and discomfort can be avoided, and patients’ autonomy can be enhanced [2–4]. In Thai and other similar Asian cultures, where diagnoses of serious illnesses are sometimes withheld from the patients by their doctors and caregivers, the estimation of short survival time also serves as a factor affecting whether the patients are allowed to know about their diagnoses [8].

Many prognostic tools to predict survival in palliative care exist [9, 10]. The PPS, one of the most studied prognostic tools in palliative care. is commonly used in Thailand [11]. The PPS was developed from the Karnofsky Performance Scale to measure physical functional performance in palliative care patients. It assesses five functional parameters, namely degree of ambulation, ability to do activities and extent of disease, ability to do self-care, food and fluid intake, and state of consciousness. PPS are in 10% decrements from 100% (fully ambulatory and healthy) to 0% (death). It is simple and practical for palliative care patients [12].

Most studies have examined the performance of the PPS for cancer patients with far fewer studies examining the performance of PPS for non-cancer patients [13]. A couple of studies have shown that PPS is associated with survival time in both cancer and non-cancer patients at the end of life, [13, 14] however, it may be less predictable in non-cancer patients [15]. Hence it has been recommended that the correlation between the PPS and survival time should be based on a local cohort where feasible [16].

This study aimed to examine the usefulness of PPS in estimating the survival time of patients with advanced cancer and non-cancer diagnoses in Chiang Mai, Thailand.

## Methods

### Participants and setting

Using a retrospective cohort design, we included all adult inpatients 18 years old or over receiving a consultation by the Palliative Care Unit at Chiang Mai University Hospital between 1 July 2018 and 31 July 2019. If a patient had more than one visit, we included information only from the first visit and excluded information for any readmission visits. Chiang Mai University Hospital is the largest university hospital in Northern Thailand and is a referral center for patients in Chiang Mai and 16 other provinces in the northern region of Thailand [17]. The institution’s consultation criteria include; negotiating an advance care plan, assisting in end-of-life care, managing socioeconomic or psychological problems, skill training for a caregiver to provide home care, and lending medical equipment for home care.

### Data collection

As part of the routine clinical assessment at the time of the first consultation, the Thai PPS was assessed by medical residents or palliative care nurses. The Thai PPS, translated from the Palliative Performance Scale version 2 (PPSv2), has been validated and is one of the most commonly used tools for palliative care assessment in Thailand. The tool shows good inter- and intra-rater reliability. The intraclass correlation coefficient (ICC) for absolute agreement is 0.911 (95% CI 0.86–0.96) and for consistency is 0.92 (95% CI 0.87–0.96). Cohen’s kappa score is 0.55 [11].

As part of routine care, patients who have been discharged after palliative care consultations should be followed up weekly (if the initial PPS ≤ 30%) or monthly (if the initial PPS > 30%). The assessment was carried out via telephone to assess patient symptoms and to give a consultation when necessary. Based on this routine follow-up, data on mortality for this study was collected up to 26 June 2020. All patients who had not died during the follow-up period were contacted on 26 June 2020.

Additional demographic information such as age, diagnoses, and co-morbidities was extracted from the hospital’s electronic medical record database. Principal diagnosis from the patient’s discharge summary was considered as the primary diagnosis while diagnoses other than the principal diagnosis were classified as co-morbidities. Uncommon co-morbidities were not recorded. Data was entered using Research Electronic Data Capture (REDCap) (https://redcap.med.cmu.ac.th), which allows data checks to minimize transcription errors (or where necessary collected on paper and later double-entered into the electronic form) and post-entry checks for extreme values. All extreme values were double-checked with the source document in the electronic medical database.

### Data analysis

Demographic characteristics and clinical data were shown as numbers and percentages for categorical variables and as mean and standard deviation for continuous variables. Baseline comparison between cancer and non-cancer patients was done using chi-square for categorical variables and t-test for continuous variables. Mann–Whitney U test was used for comparing the median number of co-morbidities between groups. The PPS at first consultation was categorized into five groups: 10%, 20%, 30%, 40–60%, and 70–80%. Survival time among cancer and non-cancer patients was demonstrated using a median, percentile, and Kaplan–Meier survival curves for all the PPS groups and between paired groups. A log-rank test was used to examine the association between the PPS and survival among cancer and non-cancer patients for all the PPS groups and between paired groups. A *p*-value of < 0.05 was considered statistically significant. All statistical analyses were performed using STATA version 15.

## Results

### Patient characteristics

Table 1 shows patient characteristics at the beginning of the research. A total of 407 patients were included in the study, 307 were cancer patients (75.4%) and 100 were non-cancer patients (24.6%). Both groups had similar proportions of males and females (45.0% of cancer patients and 49.0% of non-cancer patients, were men). The mean age of cancer patients was significantly lower than non-cancer patients, 60.6 (SD 14.9) and 70.4 (SD 18.3) years old, respectively. Almost all patients had health coverage (96.4% of cancer patients and 94% of non-cancer patients).

**Table 1:** Patient characteristics

	**Cancer** **(*n* = 307)**	**Non-cancer** **(*n* = 100)**	***P*-value**
Sex, n (%)			0.48
Male	138 (44.9)	49 (49)	
Female	169 (55.1)	51 (51)	
Age (Mean ± SD)	60.6 ± 14.9	70.4 ± 18.3	< 0.001
Age group, n (%)			< 0.001
< 45	41 (13.4)	10 (10)	
45–54	56 (18.2)	13 (13)	
55–64	84 (27.4)	15 (15)	
65–74	71 (23.1)	10 (10)	
75–84	42 (13.7)	25 (25)	
≥ 85	13 (4.2)	27 (27)	
Co-morbidities, n (%)			
Hypertension	96 (31.3)	52 (52)	< 0.001
Diabetes mellitus	50 (16.3)	23 (23)	0.13
Dyslipidemia	62 (20.2)	34 (34)	0.01
Chronic kidney disease stage II-IV	69 (22.5)	22 (22)	0.92
Psychiatric disorders	22 (7.2)	11 (11)	0.22
Number of co-morbidities, median (percentile 25, percentile 75)	1 (0, 2)	1 (0, 3)	< 0.001*
Number of co-morbidities, n (%)			0.001
none	146 (47.5)	26 (26)	
1	73 (23.8)	27 (27)	
2	45 (14.7)	20 (20)	
≥ 3	43 (14.0)	27 (27)	
Cancer types, n (%)			
Gastrointestinal	122 (39.7)		
Lung	54 (17.6)		
Genitourinary	51 (16.6)		
Breast	25 (8.1)		
Hematologic	21 (6.8)		
Bone and soft tissue	8 (2.6)		
Others	28 (8.5)		
Non-cancer diagnoses, n (%)			
Neurological disorders		38 (38)	
Heart disease		13 (13)	
End-stage renal disease		12 (12)	
Others		37 (37)	

^*^ *P*-value from Mann–Whitney U test

The three most common cancers in patients consulted by the palliative care unit were gastrointestinal, lung, and genitourinary cancers. As for non-cancer patients, the three most prevalent diagnoses were neurological disorders (including 26 strokes and 12 other neurological disorders, heart disease, and renal failure. Other diagnoses consisted of a wide range of diseases such as pneumonia, COPD (chronic obstructive pulmonary disease), HIV/AIDs (Human Immunodeficiency Virus/ Acquired immunodeficiency syndrome), and intertrochanteric fracture, none had a prevalence of over 6%. The most common co-morbidity in both cancer and non-cancer patients was hypertension. Non-cancer patients had significantly more comorbidities than cancer patients (Table 1). The top three reasons for a palliative care consultation in both groups were to negotiate an advance care plan (87.6% of cancer patients and 66% of non-cancer patients), to assist in end-of-life care (36.5% of cancer patients and 49% of non-cancer patients) and to manage socioeconomic or psychological problems (20.2% of cancer patients and 17% of non-cancer patients). Most patients had a PPS of $\le$ 30% (58.3% for cancer patients and 89% for non-cancer patients). The distribution of PPS groups between cancer and non-cancer patients differed significantly (*p*-value< 0.001) with a higher PPS found among cancer patients than non-cancer patients, as only cancer patients received palliative care consultation at PPS 70–80%. The proportion of patients with PPS 40–60% were 39.1% in cancer patients and 11% in non-cancer patients (Table 2).

**Table 2:** PPS of cancer and non-cancer patients

PPS (%)	Cancer patients n (%) (*n* = 307)	Non-cancer patients n (%) (*n* = 100)
10	24 (7.8)	28 (28)
20	39 (12.7)	14 (14)
30	116 (37.8)	47 (47)
40	61 (19.9)	6 (6)
50	35 (11.4)	4 (4)
60	22 (7.2)	1 (1)
70	5 (1.6)	0 (0)
80	5 (1.6)	0 (0)

*PPS* Palliative Performance Scale

### PPS and survival time

**Fig. 1:**
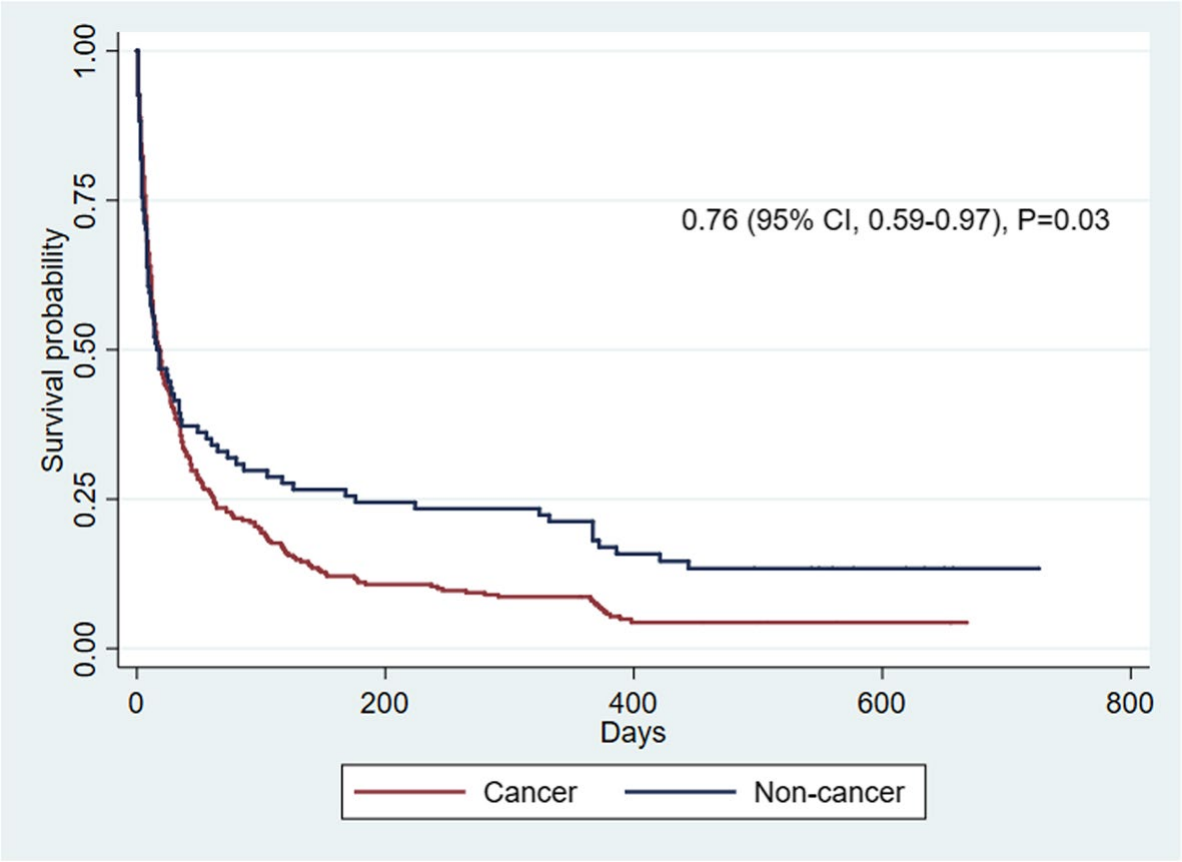
Kaplan–Meier survival curves in patients with cancer and non-cancer diagnoses

**Fig. 2:**
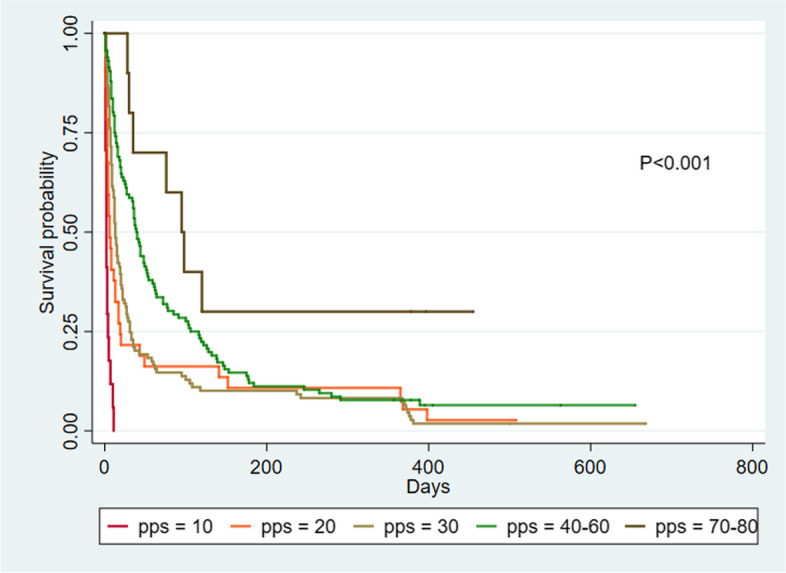
Kaplan–Meier survival curves by Palliative Performance Scale in patients with cancer diagnoses

**Fig. 3:**
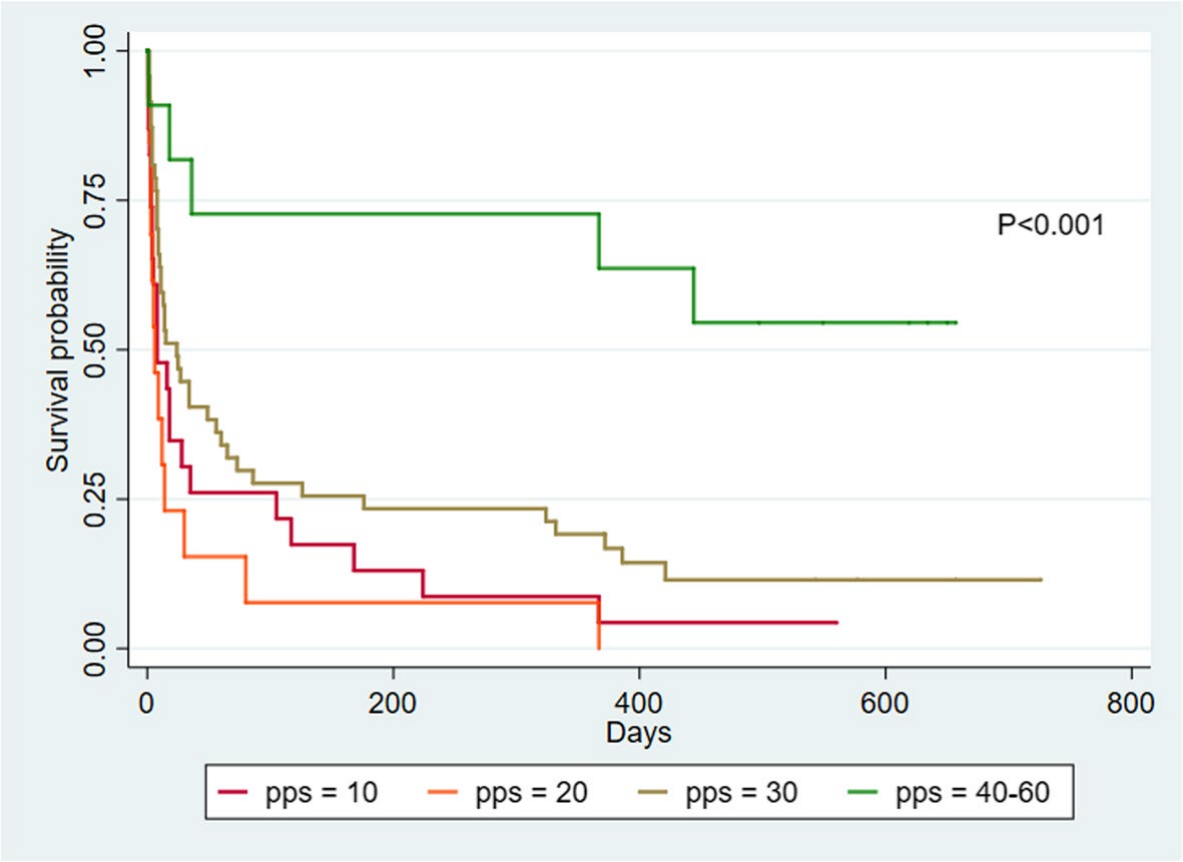
Kaplan–Meier survival curves by Palliative Performance Scale in patients with non-cancer diagnoses

Kaplan–Meier survival curves and log-rank tests comparing survival time between both groups of patients showed that non-cancer patients had a significantly longer survival time than cancer patients (*p*-value 0.03). Cox-regression showed hazard ratio of 0.76 with 95% confidence interval of 0.59 to 0.97, *p*-value 0.03 (Fig. 1). The PPS was associated with survival time among cancer (*p*-value< 0.001) and non-cancer patients (*p*-value< 0.001). (Figs. 2 and 3) Log-rank test between PPS 10–30% and 40–60% showed *p*-value< 0.001 in cancer patients (Fig. 2).

By contrast, the median survival time based on PPS differed between cancer and non-cancer patients. For cancer patients, there was an association between PPS and median survival time. The median survival times [percentile 25, percentile 75] for those with PPS of 10, 20, 30 were 2 days [1, 4], 6 days [3, 19] and 13 days [7, 31], respectively. In addition, those with PPS of 40–60 and 70–80 were 39 days [12, 106] and 95 days [35], respectively (Table 3). Most pairwise comparisons in cancer patients between PPS 10 vs. 20% (*p*-value < 0.001), PPS 30 vs. 40–60% (*p*-value < 0.01) and PPS 40–60 vs. 70–80% (*p*-value 0.05) showed significant differences except for PPS 20 vs. 30% (*p*-value 0.29).

**Table 3:** Median survival time of the palliative care patients

	**Median survival time,** **days [percentile 25, percentile 75]**	
**PPS (%)**	**Cancer patients** **(*n* = 307)**	**Non-cancer patients** **(*n* = 100)**
10	2 [1, 4]	8 [3, 105]
20	6 [3, 19]	6 [3, 14]
30	13 [7, 31]	24 [8, 176]
40–60	39 [12, 106]	-
70–80	95 [35,.]	-
Total	18 [5, 58]	16 [5, 176]

*PPS* Palliative Performance Scale

For non-cancer patients, the logical association between PPS and median survival time was not observed. The median survival time [percentile 25, percentile 75] for those with PPS of 10, 20, and 30% were 8 days [3, 105], 6 days [3, 14], and 24 days [8, 176] respectively. The sample size for non-cancer patients with PPS 40–60% was small (*n* = 11) and the median survival time was not reached (Table 3). Similarly, pairwise comparisons in non-cancer patients between PPS 20 vs. 30%; and PPS 30 vs. 40–60% showed significant differences (*p*-value 0.02 and 0.004 respectively) but did not show significant differences between PPS 10 vs. 20% (*p*-value 0.38). Significant difference between survival time in non-cancer patients with PPS 10–30% and 40–60% were seen (*p*-value < 0.001).

## Discussion

This study suggests that a low PPS ($\le$ 30%) can serve as a predictor of the last months of life in both cancer and non-cancer patients. The study helps confirm that the PPS is a reliable prognosticator in estimating survival time for cancer patients but due to unexpected findings of PPS 10 and 20% in non-cancer patients, further studies are required to conclude the reliability of PPS in predicting survival time in non-cancer patients.

The median survival times for cancer patients with a PPS of 10, 20, 30, 40–60, and 70–80% were 2, 6, 13, 39, and 95 days, respectively. This is in line with previous studies for cancer patients where death was expected within: days if the PPS were 10%; a week if the PPS was 20%, weeks if the PPS was 30% and within months if PPS was over 40% [13]. However, there are still slight variations potentially due to some differences in standards of care in different settings. A study in a home-based hospice and palliative care program showed a median survival of 3, 5, 10, 19, 29.5, 35 days for PPS 10 to 60%, respectively [18]. One study in patients admitted to a palliative care unit in South Korea showed initial PPS after palliative care consultation of 10–20%, 30–50%, and more than 60% to have a median survival time of 14, 19, and 38 days [19]. Another study, in a Southeast Asian population who were admitted to a palliative care unit in a tertiary hospital found the mean survival time to be 19 and 43 days in patients at PPS of 10–30% and 40–60% [20].

In non-cancer patients, patients with a PPS of 10–30% had a significantly lower survival time compared to a PPS of 40–60%. The association for survival was not logical among the non-cancer patients with very low-performance status (PPS 10–20%). Many of the non-cancer patients with PPS 40–60% and even some of the patients with PPS ≤ 30% may have prolonged survival, despite the need for palliative care consultation. This cohort also had a wider variation in survival time than cancer patients. A large proportion of non-cancer patients in our study had a diagnosis of cerebrovascular disease (stroke), common in South-East Asian countries [21]. Stroke may lead to low-performance status but may still be associated with a relatively long survival. Thus, low PPS in stroke patients is not necessarily associated with short survival time [22]. Another explanation why the theoretical gradient relationship between lower PPS and shorter survival time was not demonstrated is likely due to the other forms of disease trajectories [22, 23]. For example, a PPS at a consultation for patients with severe end-organ failures presenting with an acute exacerbation may not fully represent actual physical functional performance. This results in an initially low PPS but a longer than expected survival time.

### Limitations

There are at least three limitations to this research. Firstly, the number of non-cancer patients was relatively small, we were not able to do a subgroup analysis of specific diseases in non-cancer patients. However, we believe that reporting a combined median survival time in non-cancer diagnoses still yields meaningful estimations for generalist palliative care providers. Secondly, data collection was done from one study site, Chiang Mai University Hospital. The patient consulted by the palliative care unit in a tertiary hospital, may have more complex diseases and/or more complex palliative care needs when compared to other settings. However, we believe that our results are still generalizable in the cancer population in Thailand, as the most common cancer types in this study resemble cancer prevalence in the country [24]. Lastly, palliative care consultations in our population occurred late in the course of disease partly due to the institution’s consultation criteria of palliative care being for end of life care only. Patients were predominantly at PPS of 10–50%, whereas PPS of 60% or more were relatively few. Thus, it was not possible to get reliable estimates for the survival time among patients with higher PPS, especially for non-cancer patients.

## Conclusion

The PPS is associated with survival time among cancer and non-cancer patients in Thailand. While PPS was a reliable predictor of survival time in advanced cancer patients, in non-cancer patients with PPS 10–30%, other considerations were needed for the prediction of survival time such as disease diagnoses. Further study should have an emphasis on prognostication in non-cancer patients to develop a more reliable tool.

## Supplementary Information

**Additional file 1.**

## Data Availability

The datasets generated during and analyzed during the current study are not publicly available as they were not part of the consent but are available from the corresponding author on reasonable request.

## Abbreviation

PPS: Palliative Performance Scale
