# Supplementary material for: Palliative Performance Scale and survival in patients with cancer and non-cancer diagnoses needing a palliative care consultation: a retrospective cohort study
Source: BMC Palliat Care. 2021 May 26;20:74. doi: 10.1186/s12904-021-00773-8 (PMC8157447; doi:10.1186/s12904-021-00773-8)

**Appendix**

**Palliative Performance Scale and survival in patients with cancer and non-cancer diagnoses needing a palliative care consultation:** **A retrospective cohort study**

Patcharaporn Prompantakorn^1^, Chaisiri Angkurawaranon^1^, Kanokporn Pinyopornpanish^1^, Lalita Chutarattanakul^1^, Chanchanok Aramrat^1^, Chanapat Pateekhum^1^, Nisachol Dejkriengkraikul^1^

*Correspondence: [namfa_22@hotmail.com](mailto:namfa_22@hotmail.com)

^1^Department of Family Medicine, Faculty of Medicine, Chiang Mai University, Chiang Mai, Thailand

**Table 1** Additional non-cancer diagnoses

| Non-cancer diagnoses | | N=100 | (%) |
| --- | --- | --- | --- |
| **Neurological disorders** | | **38** | **(38)** |
|  | Stroke | 26 | (26) |
|  | Hypoxic-ischemic encephalopathy | 4 | (4) |
|  | High cervical nerves spinal cord injury | 2 | (2) |
|  | advanced dementia | 2 | (2) |
|  | Seizure and epilepsy | 2 | (2) |
|  | Meningoencephalitis | 2 | (2) |
| **Heart disease** | | **13** | **(13)** |
| **End stage renal disease** | | **12** | **(12)** |
| **Others** | | **37** | **(37)** |
|  | Pneumonia | 6 | (5) |
|  | Chronic obstructive pulmonary disease | 5 | (5) |
|  | HIV/AIDs | 4 | (4) |
|  | Intertrochanteric fracture | 4 | (4) |
|  | Chronic myelitis | 3 | (3) |
|  | Diffused systemic sclerosis | 3 | (3) |
|  | Multiple trauma | 3 | (3) |
|  | Upper gastrointestinal hemorrhage | 2 | (2) |
|  | Asthma | 2 | (2) |
|  | Sepsis from infected wound | 2 | (2) |
|  | Sepsis (unknown source of infection) | 2 | (2) |
|  | Abdominal aortic aneurysm | 1 | (1) |

HIV/AIDs: Human Immunodeficiency Virus/Acquired immunodeficiency syndrome

**Figure 1** Pairwise comparison between PPS 10 and 20% in cancer patients


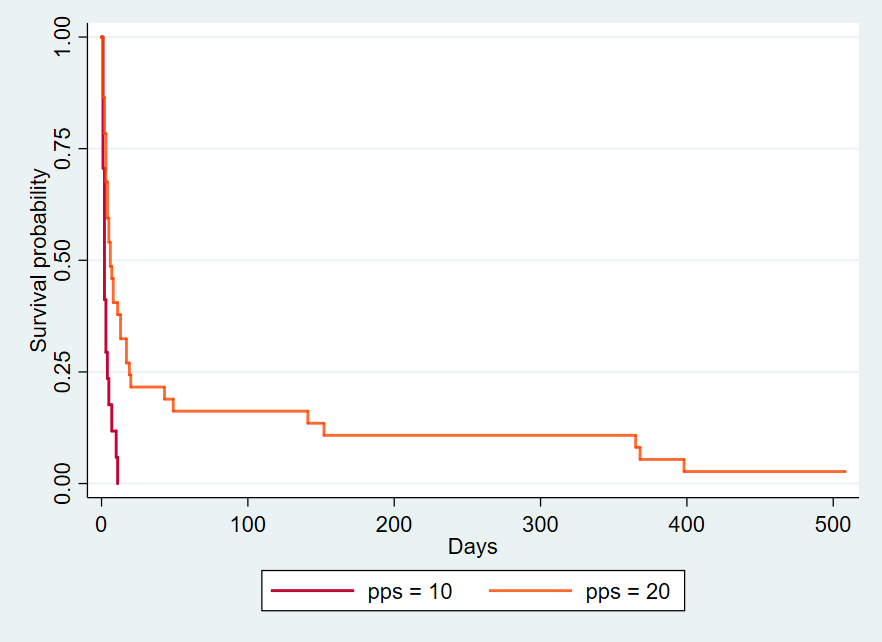


**Figure 2** Pairwise comparison between PPS 20 and 30% in cancer patients


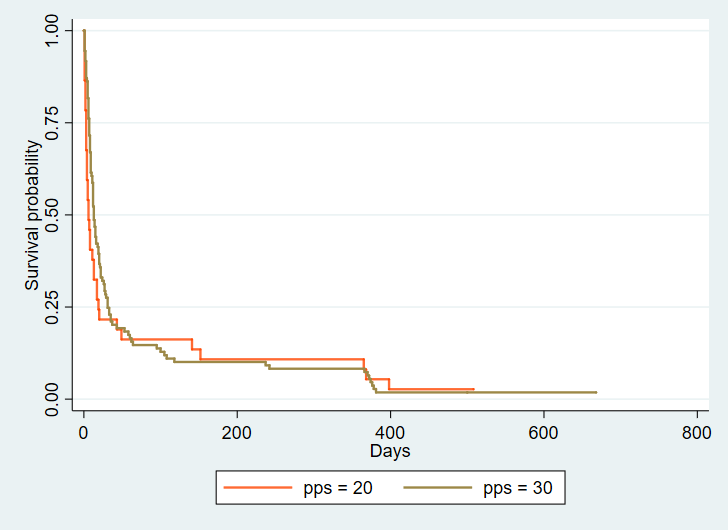


**Figure 3** Pairwise comparison between PPS 30 and 40-60% in cancer patients


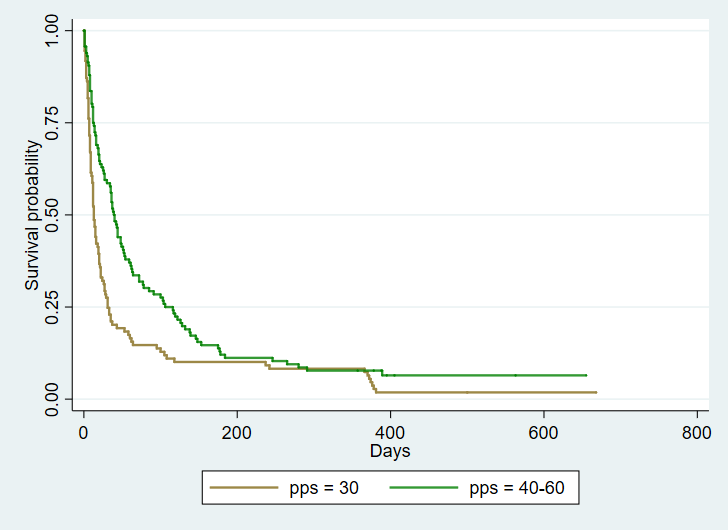


**Figure 4** Pairwise comparison between PPS 40-60 and 70-80% in cancer patients


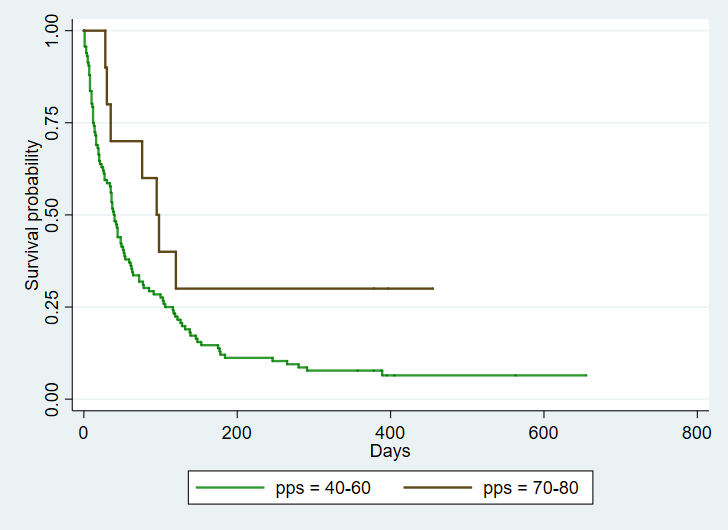


**Figure 5** Pairwise comparison between PPS 10 and 20% in non-cancer patients


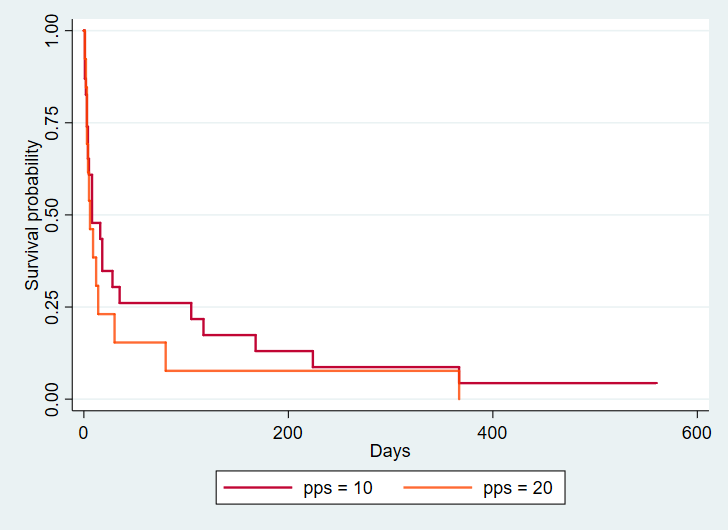


**Figure 6** Pairwise comparison between PPS 20 and 30% in non-cancer patients


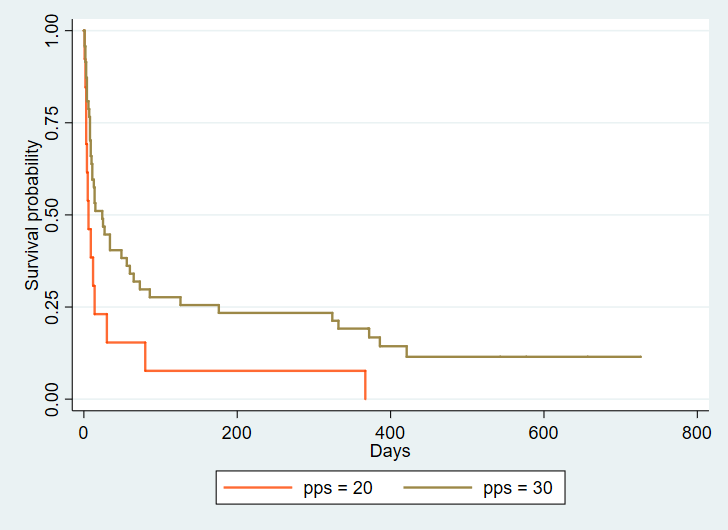


**Figure 7** Pairwise comparison between PPS 30 and 40-60% in non-cancer patients


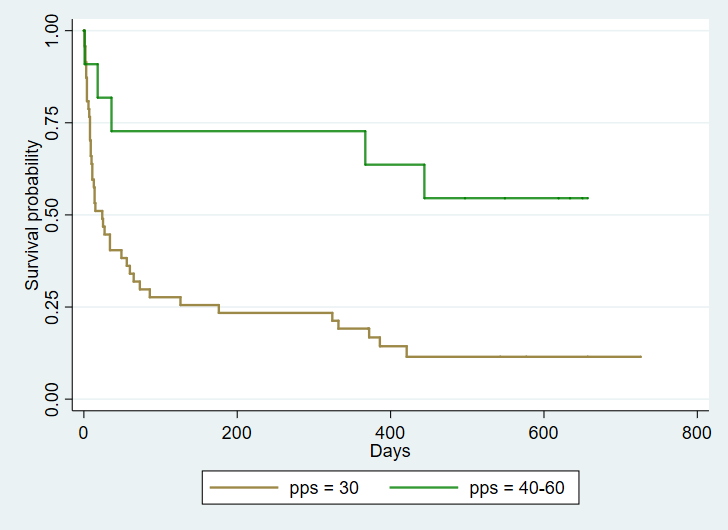


**Figure 8** Comparison between PPS 10-30 and 40-60% in non-cancer patients


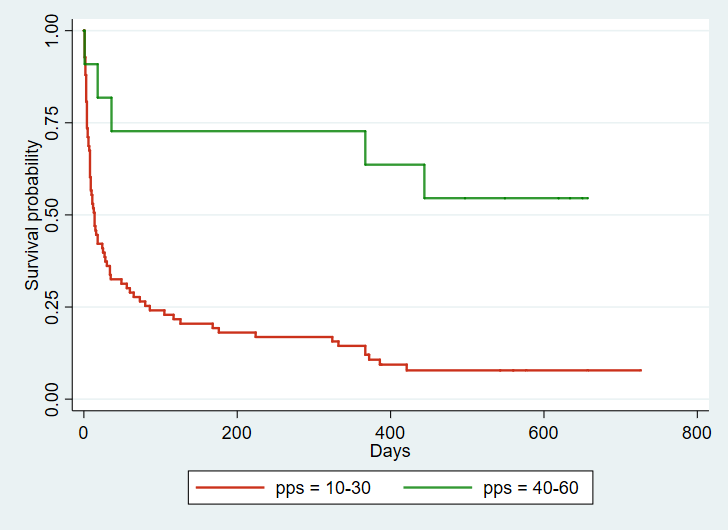

Supplement: Supplementary file 1 — Additional file 1. [file 12904_2021_773_MOESM1_ESM.docx]
